# OA16.03. Patients seek integrative medicine for preventive approach to health

**DOI:** 10.1186/1472-6882-12-S1-O64

**Published:** 2012-06-12

**Authors:** R Wolever

**Affiliations:** 1 Duke Integrative Medicine, Durham, USA

## Purpose

Integrative medicine (IM) has emerged as a new discipline with its own specialty centers, fellowship programs, and certification processes. However, the reasons patients seek IM have not been well characterized in the literature. This is the first large-scale study in IM designed to systematically characterize patients seeking care at IM clinics.

## Methods

The BraveNet practice-based research network enrolled 3940 eligible participants who were receiving treatment by a clinician at one of 8 IM clinics. Within 2 weeks of their IM clinic visit, participants provided demographics and lifestyle information, and ranked treatment goals and reasons for seeking care at an IM clinic. Clinicians documented the medical condition treated and services provided. A central database received de-identified data through a secure website. All sites and the coordinating center received IRB approval.

## Results

Top-ranked reasons were the desire to: (1) improve health and wellness now to prevent future problems (83.8%); (2) try new options for health care (78.6%); and (3) maximize health regardless of whether or not illness is curable (73.8%). When analyzed separately by sex and patient status (new versus returning), the top 2 reasons remained the same. Fourteen of the 17 most common health condition subgroups reported “the desire to … prevent future problems” as the most important reason for seeking IM. Additional findings include sociodemographics, medical conditions addressed, lifestyle-related behaviors (including exercise, tobacco and alcohol use, weight), patient goals, and the frequencies of therapeutic services most commonly provided.

## Conclusion

Our findings represent an important step in IM research as the first large scale study to characterize patients seen across multiple IM clinics. Patients at IM centers desire to expand the current paradigm of health care, seeking preventive and novel options consistent with those proposed in the current U.S. health care reform effort.

